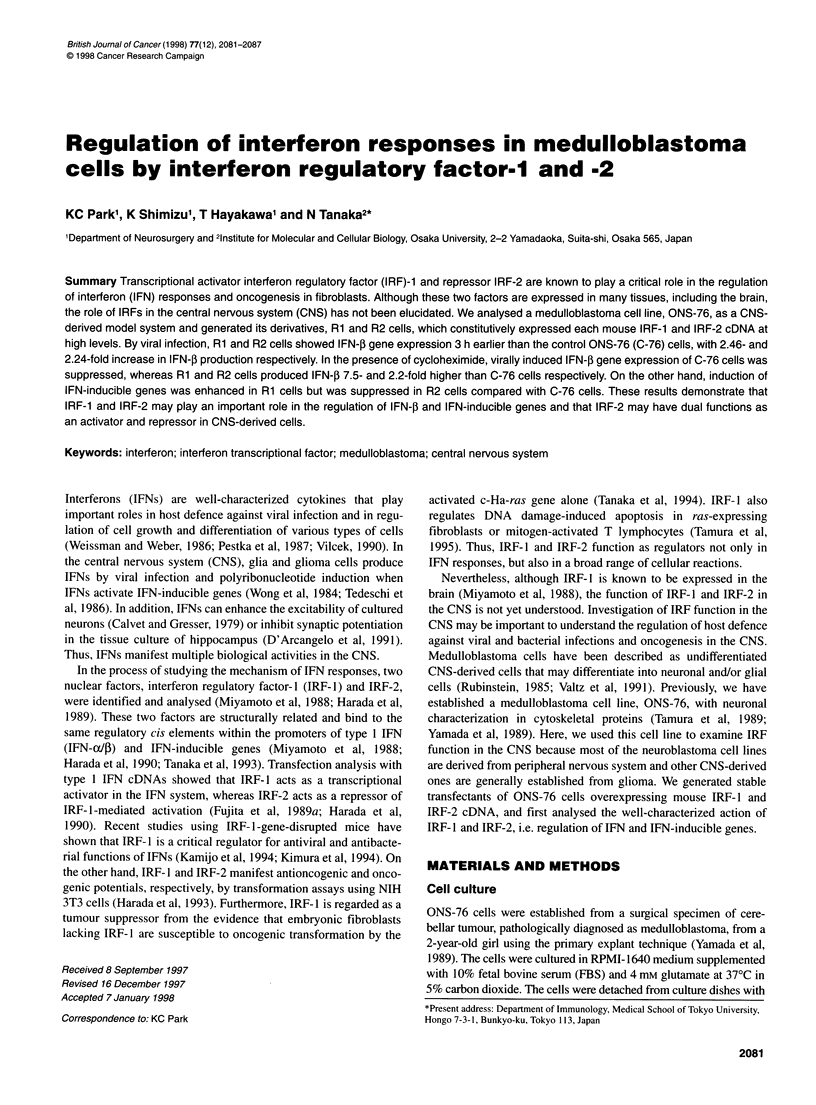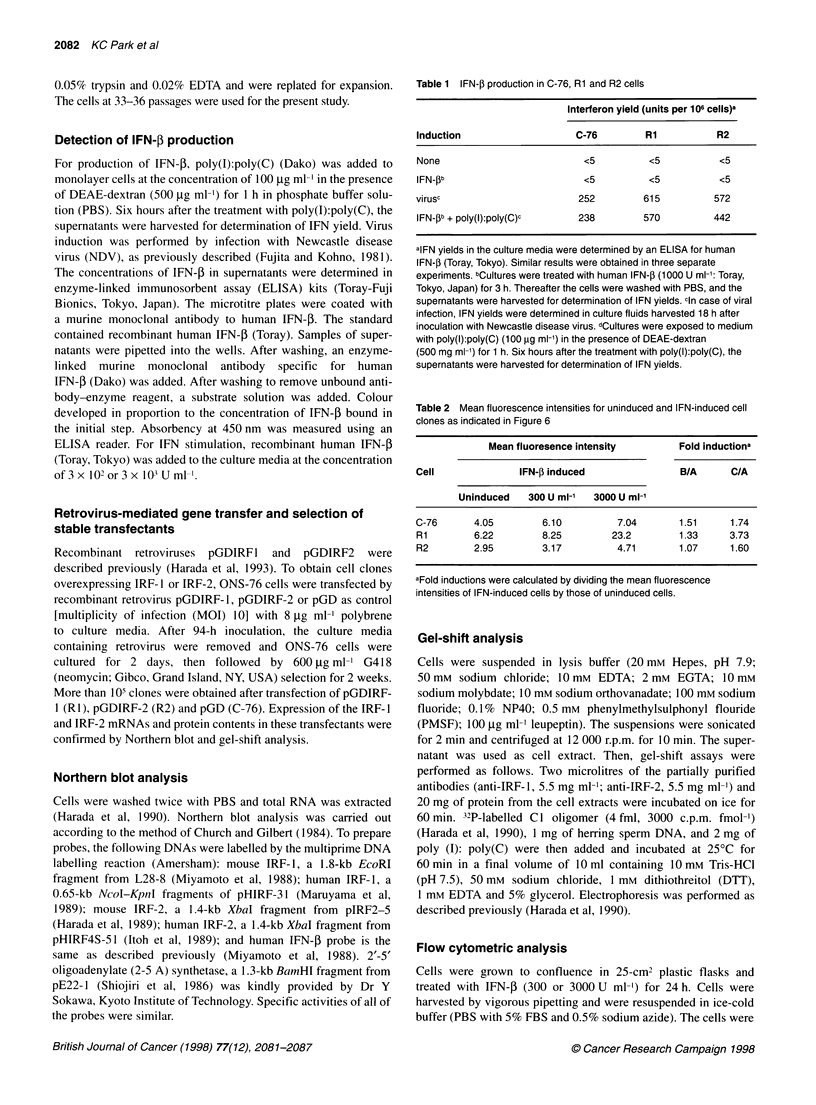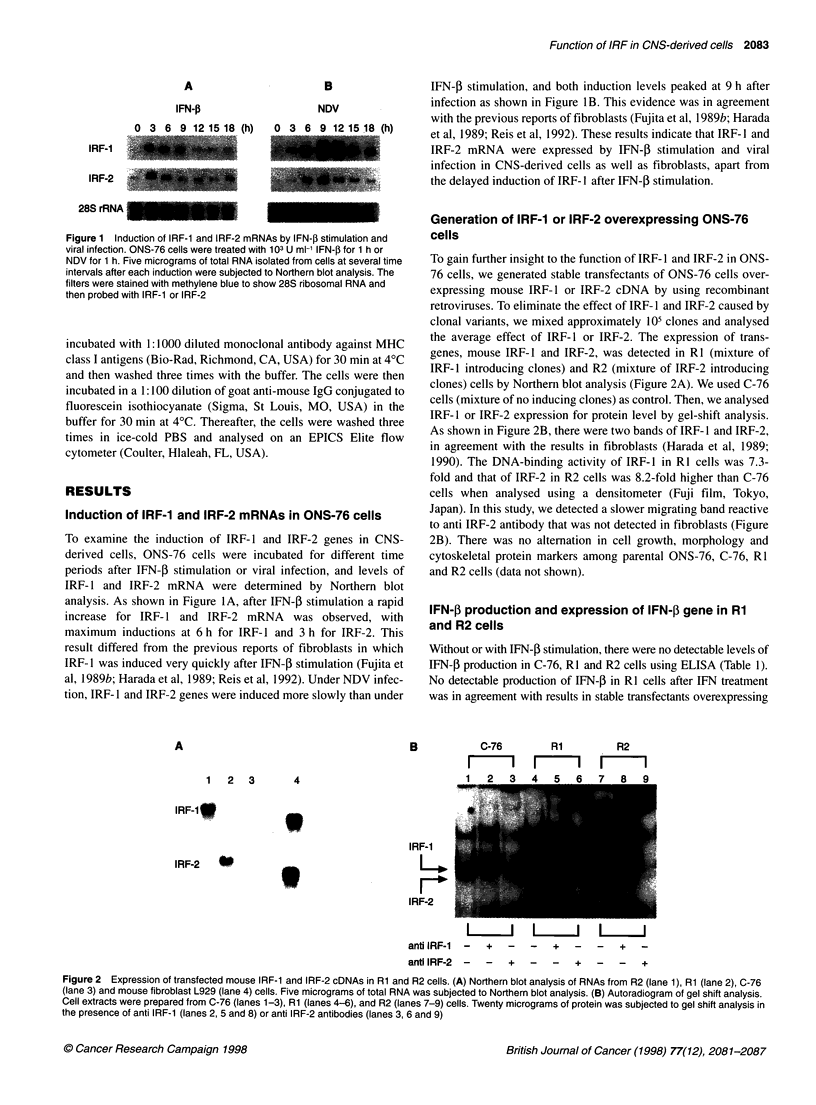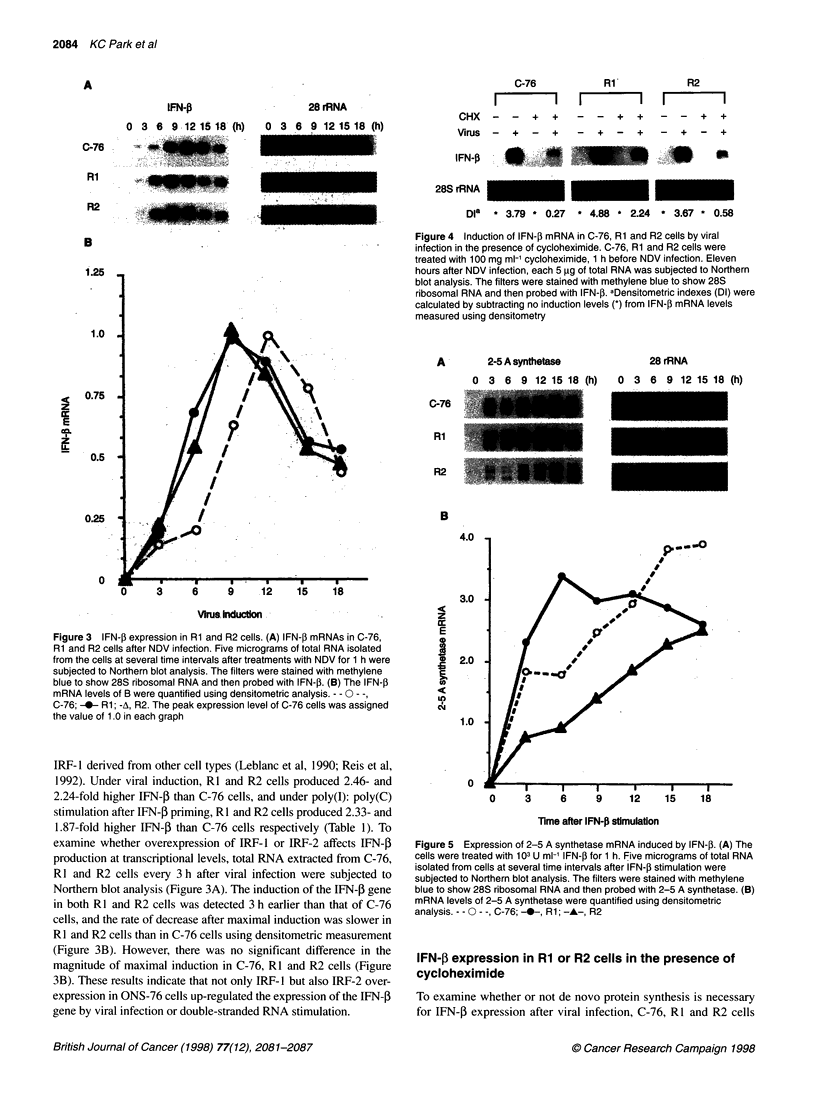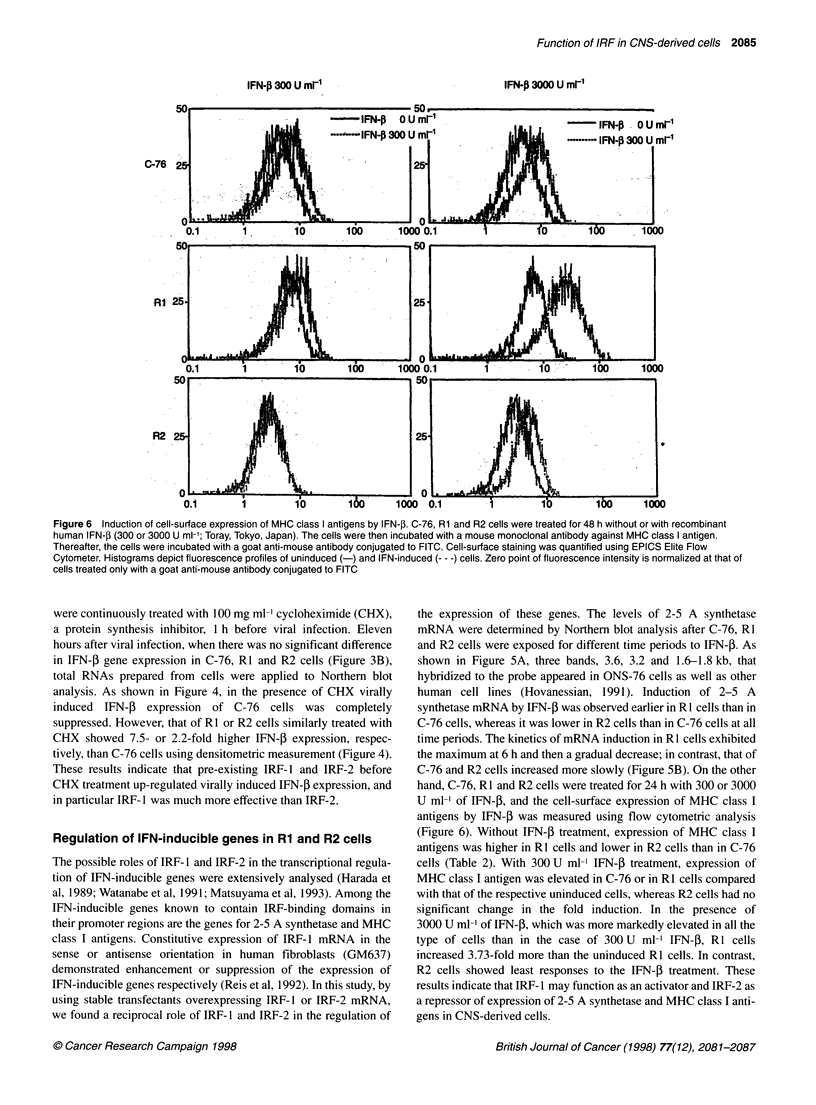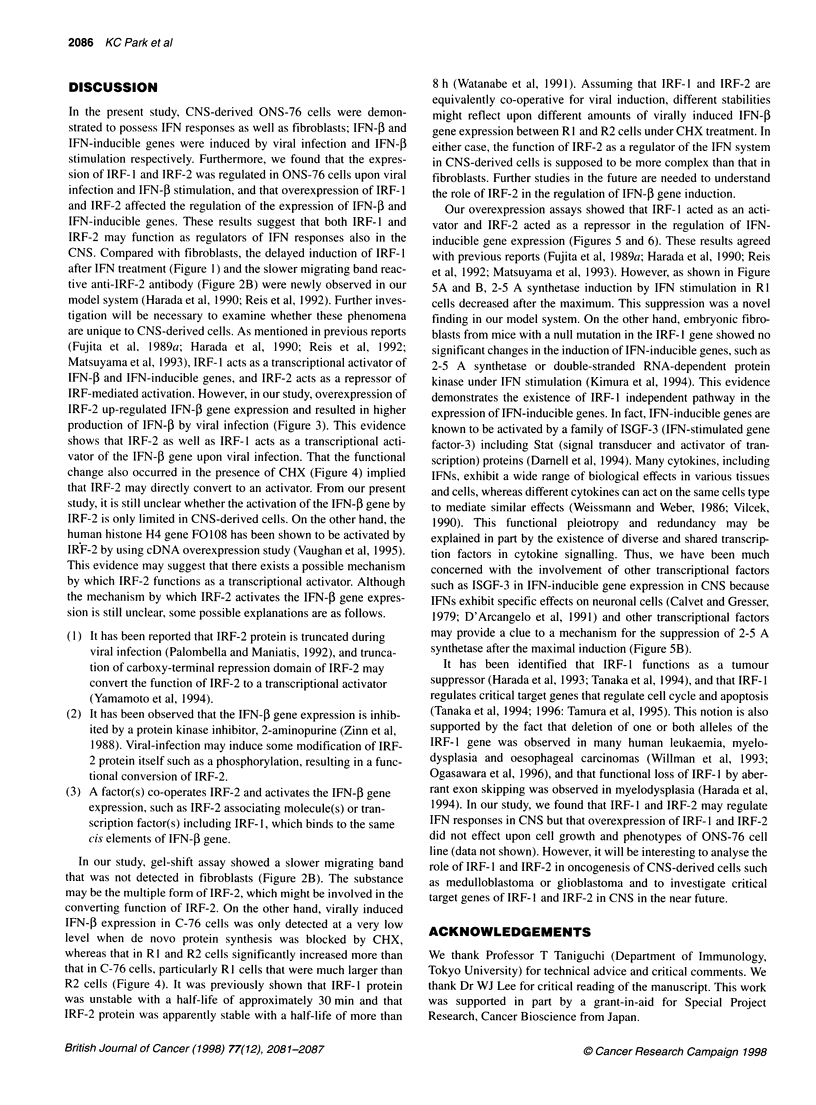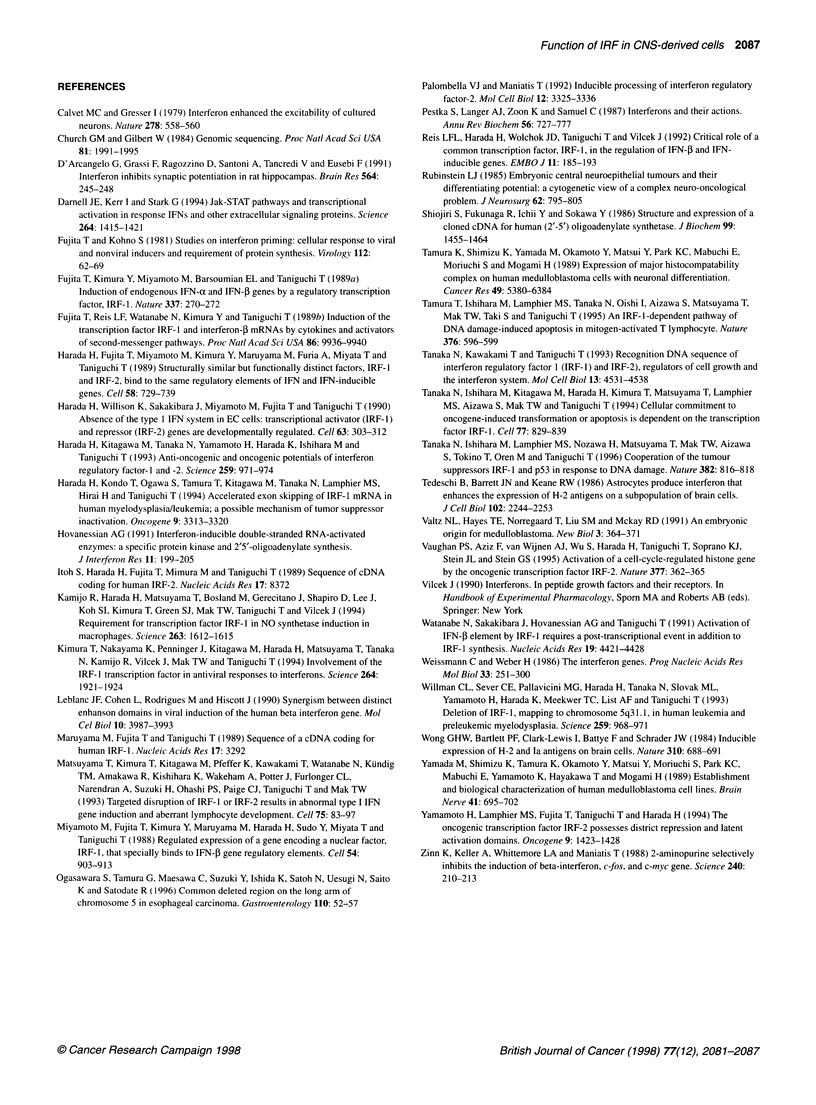# Regulation of interferon responses in medulloblastoma cells by interferon regulatory factor-1 and -2.

**DOI:** 10.1038/bjc.1998.351

**Published:** 1998-06

**Authors:** K. C. Park, K. Shimizu, T. Hayakawa, N. Tanaka

**Affiliations:** Department of Neurosurgery, Osaka University, Japan.

## Abstract

**Images:**


British Journal of Cancer (1998) 77(12), 2081–2087
© 1998 Cancer Research Campaign

### Regulation of interferon responses in medulloblastoma cells by interferon regulatory factor-1 and -2

KC Park[1], K Shimizu[1], T Hayakawa[1] and N Tanaka[2]*

[1]Department of Neurosurgery and [2]Institute for Molecular and Cellular Biology, Osaka University, 2-2 Yamadaoka, Suita-shi, Osaka 565, Japan

**Summary** Transcriptional activator interferon regulatory factor (IRF)-1 and repressor IRF-2 are known to play a critical role in the regulation of interferon (IFN) responses and oncogenesis in fibroblasts. Although these two factors are expressed in many tissues, including the brain, the role of IRFs in the central nervous system (CNS) has not been elucidated. We analysed a medulloblastoma cell line, ONS-76, as a CNS-derived model system and generated its derivatives, R1 and R2 cells, which constitutively expressed each mouse IRF-1 and IRF-2 cDNA at high levels. By viral infection, R1 and R2 cells showed IFN-β gene expression 3 h earlier than the control ONS-76 (C-76) cells, with 2.46- and 2.24-fold increase in IFN-β production respectively. In the presence of cycloheximide, virally induced IFN-β gene expression of C-76 cells was suppressed, whereas R1 and R2 cells produced IFN-β 7.5- and 2.2-fold higher than C-76 cells respectively. On the other hand, induction of IFN-inducible genes was enhanced in R1 cells but was suppressed in R2 cells compared with C-76 cells. These results demonstrate that IRF-1 and IRF-2 may play an important role in the regulation of IFN-β and IFN-inducible genes and that IRF-2 may have dual functions as an activator and repressor in CNS-derived cells.

**Keywords:** interferon; interferon transcriptional factor; medulloblastoma; central nervous system

Interferons (IFNs) are well-characterized cytokines that play important roles in host defence against viral infection and in regulation of cell growth and differentiation of various types of cells (Weissman and Weber, 1986; Pestka et al, 1987; Vilcek, 1990). In the central nervous system (CNS), glia and glioma cells produce IFNs by viral infection and polyribonucleotide induction when IFNs activate IFN-inducible genes (Wong et al, 1984; Tedeschi et al, 1986). In addition, IFNs can enhance the excitability of cultured neurons (Calvet and Gresser, 1979) or inhibit synaptic potentiation in the tissue culture of hippocampus (D'Arcangelo et al, 1991). Thus, IFNs manifest multiple biological activities in the CNS.

In the process of studying the mechanism of IFN responses, two nuclear factors, interferon regulatory factor-1 (IRF-1) and IRF-2, were identified and analysed (Miyamoto et al, 1988; Harada et al, 1989). These two factors are structurally related and bind to the same regulatory *cis* elements within the promoters of type 1 IFN (IFN-α/β) and IFN-inducible genes (Miyamoto et al, 1988; Harada et al, 1990; Tanaka et al, 1993). Transfection analysis with type 1 IFN cDNAs showed that IRF-1 acts as a transcriptional activator in the IFN system, whereas IRF-2 acts as a repressor of IRF-1-mediated activation (Fujita et al, 1989*a*; Harada et al, 1990). Recent studies using IRF-1-gene-disrupted mice have shown that IRF-1 is a critical regulator for antiviral and antibacterial functions of IFNs (Kamijo et al, 1994; Kimura et al, 1994). On the other hand, IRF-1 and IRF-2 manifest antioncogenic and oncogenic potentials, respectively, by transformation assays using NIH 3T3 cells (Harada et al, 1993). Furthermore, IRF-1 is regarded as a tumour suppressor from the evidence that embryonic fibroblasts lacking IRF-1 are susceptible to oncogenic transformation by the activated c-Ha-*ras* gene alone (Tanaka et al, 1994). IRF-1 also regulates DNA damage-induced apoptosis in *ras*-expressing fibroblasts or mitogen-activated T lymphocytes (Tamura et al, 1995). Thus, IRF-1 and IRF-2 function as regulators not only in IFN responses, but also in a broad range of cellular reactions.

Nevertheless, although IRF-1 is known to be expressed in the brain (Miyamoto et al, 1988), the function of IRF-1 and IRF-2 in the CNS is not yet understood. Investigation of IRF function in the CNS may be important to understand the regulation of host defence against viral and bacterial infections and oncogenesis in the CNS. Medulloblastoma cells have been described as undifferentiated CNS-derived cells that may differentiate into neuronal and/or glial cells (Rubinstein, 1985; Valtz et al, 1991). Previously, we have established a medulloblastoma cell line, ONS-76, with neuronal characterization in cytoskeletal proteins (Tamura et al, 1989; Yamada et al, 1989). Here, we used this cell line to examine IRF function in the CNS because most of the neuroblastoma cell lines are derived from peripheral nervous system and other CNS-derived ones are generally established from glioma. We generated stable transfectants of ONS-76 cells overexpressing mouse IRF-1 and IRF-2 cDNA, and first analysed the well-characterized action of IRF-1 and IRF-2, i.e. regulation of IFN and IFN-inducible genes.

### MATERIALS AND METHODS

### Cell culture

ONS-76 cells were established from a surgical specimen of cerebellar tumour, pathologically diagnosed as medulloblastoma, from a 2-year-old girl using the primary explant technique (Yamada et al, 1989). The cells were cultured in RPMI-1640 medium supplemented with 10% fetal bovine serum (FBS) and 4 mM glutamate at 37°C in 5% carbon dioxide. The cells were detached from culture dishes with

*Received 8 September 1997*
*Revised 16 December 1997*
*Accepted 7 January 1998*

*Correspondence to:* KC Park

*Present address: Department of Immunology, Medical School of Tokyo University, Hongo 7-3-1, Bunkyo-ku, Tokyo 113, Japan

0.05% trypsin and 0.02% EDTA and were replated for expansion. The cells at 33–36 passages were used for the present study.

### Detection of IFN-β production

For production of IFN-β, poly(I):poly(C) (Dako) was added to monolayer cells at the concentration of 100 μg ml$^{-1}$ in the presence of DEAE-dextran (500 μg ml$^{-1}$) for 1 h in phosphate buffer solution (PBS). Six hours after the treatment with poly(I):poly(C), the supernatants were harvested for determination of IFN yield. Virus induction was performed by infection with Newcastle disease virus (NDV), as previously described (Fujita and Kohno, 1981). The concentrations of IFN-β in supernatants were determined in enzyme-linked immunosorbent assay (ELISA) kits (Toray-Fuji Bionics, Tokyo, Japan). The microtitre plates were coated with a murine monoclonal antibody to human IFN-β. The standard contained recombinant human IFN-β (Toray). Samples of supernatants were pipetted into the wells. After washing, an enzyme-linked murine monoclonal antibody specific for human IFN-β (Dako) was added. After washing to remove unbound antibody–enzyme reagent, a substrate solution was added. Colour developed in proportion to the concentration of IFN-β bound in the initial step. Absorbency at 450 nm was measured using an ELISA reader. For IFN stimulation, recombinant human IFN-β (Toray, Tokyo) was added to the culture media at the concentration of $3 \times 10^2$ or $3 \times 10^3$ U ml$^{-1}$.

### Retrovirus-mediated gene transfer and selection of stable transfectants

Recombinant retroviruses pGDIRF1 and pGDIRF2 were described previously (Harada et al, 1993). To obtain cell clones overexpressing IRF-1 or IRF-2, ONS-76 cells were transfected by recombinant retrovirus pGDIRF-1, pGDIRF-2 or pGD as control [multiplicity of infection (MOI) 10] with 8 μg ml$^{-1}$ polybrene to culture media. After 94-h inoculation, the culture media containing retrovirus were removed and ONS-76 cells were cultured for 2 days, then followed by 600 μg ml$^{-1}$ G418 (neomycin; Gibco, Grand Island, NY, USA) selection for 2 weeks. More than $10^5$ clones were obtained after transfection of pGDIRF-1 (R1), pGDIRF-2 (R2) and pGD (C-76). Expression of the IRF-1 and IRF-2 mRNAs and protein contents in these transfectants were confirmed by Northern blot and gel-shift analysis.

### Northern blot analysis

Cells were washed twice with PBS and total RNA was extracted (Harada et al, 1990). Northern blot analysis was carried out according to the method of Church and Gilbert (1984). To prepare probes, the following DNAs were labelled by the multiprime DNA labelling reaction (Amersham): mouse IRF-1, a 1.8-kb *Eco*RI fragment from L28-8 (Miyamoto et al, 1988); human IRF-1, a 0.65-kb *Nco*I–*Kpn*I fragments of pHIRF-31 (Maruyama et al, 1989); mouse IRF-2, a 1.4-kb *Xba*I fragment from pIRF2–5 (Harada et al, 1989); human IRF-2, a 1.4-kb *Xba*I fragment from pHIRF4S-51 (Itoh et al, 1989); and human IFN-β probe is the same as described previously (Miyamoto et al, 1988). 2′-5′ oligoadenylate (2-5 A) synthetase, a 1.3-kb *Bam*HI fragment from pE22-1 (Shiojiri et al, 1986) was kindly provided by Dr Y Sokawa, Kyoto Institute of Technology. Specific activities of all of the probes were similar.

**Table 1** IFN-β production in C-76, R1 and R2 cells

| | Interferon yield (units per $10^6$ cells)[a] | | |
|---|---|---|---|
| **Induction** | **C-76** | **R1** | **R2** |
| None | <5 | <5 | <5 |
| IFN-β[b] | <5 | <5 | <5 |
| virus[c] | 252 | 615 | 572 |
| IFN-β[b] + poly(I):poly(C)[c] | 238 | 570 | 442 |

[a]IFN yields in the culture media were determined by an ELISA for human IFN-β (Toray, Tokyo). Similar results were obtained in three separate experiments. [b]Cultures were treated with human IFN-β (1000 U ml$^{-1}$: Toray, Tokyo, Japan) for 3 h. Thereafter the cells were washed with PBS, and the supernatants were harvested for determination of IFN yields. [c]In case of viral infection, IFN yields were determined in culture fluids harvested 18 h after inoculation with Newcastle disease virus. [d]Cultures were exposed to medium with poly(I):poly(C) (100 μg ml$^{-1}$) in the presence of DEAE-dextran (500 mg ml$^{-1}$) for 1 h. Six hours after the treatment with poly(I):poly(C), the supernatants were harvested for determination of IFN yields.

**Table 2** Mean fluorescence intensities for uninduced and IFN-induced cell clones as indicated in Figure 6

| | Mean fluoresence intensity | | | Fold induction[a] | |
|---|---|---|---|---|---|
| **Cell** | | **IFN-β induced** | | **B/A** | **C/A** |
| | **Uninduced** | **300 U ml$^{-1}$** | **3000 U ml$^{-1}$** | | |
| C-76 | 4.05 | 6.10 | 7.04 | 1.51 | 1.74 |
| R1 | 6.22 | 8.25 | 23.2 | 1.33 | 3.73 |
| R2 | 2.95 | 3.17 | 4.71 | 1.07 | 1.60 |

[a]Fold inductions were calculated by dividing the mean fluorescence intensities of IFN-induced cells by those of uninduced cells.

### Gel-shift analysis

Cells were suspended in lysis buffer (20 mM Hepes, pH 7.9; 50 mM sodium chloride; 10 mM EDTA; 2 mM EGTA; 10 mM sodium molybdate; 10 mM sodium orthovanadate; 100 mM sodium fluoride; 0.1% NP40; 0.5 mM phenylmethylsulphonyl flouride (PMSF); 100 μg ml$^{-1}$ leupeptin). The suspensions were sonicated for 2 min and centrifuged at 12 000 r.p.m. for 10 min. The supernatant was used as cell extract. Then, gel-shift assays were performed as follows. Two microlitres of the partially purified antibodies (anti-IRF-1, 5.5 mg ml$^{-1}$; anti-IRF-2, 5.5 mg ml$^{-1}$) and 20 mg of protein from the cell extracts were incubated on ice for 60 min. $^{32}$P-labelled C1 oligomer (4 fml, 3000 c.p.m. fmol$^{-1}$) (Harada et al, 1990), 1 mg of herring sperm DNA, and 2 mg of poly (I): poly(C) were then added and incubated at 25°C for 60 min in a final volume of 10 ml containing 10 mM Tris-HCl (pH 7.5), 50 mM sodium chloride, 1 mM dithiothreitol (DTT), 1 mM EDTA and 5% glycerol. Electrophoresis was performed as described previously (Harada et al, 1990).

### Flow cytometric analysis

Cells were grown to confluence in 25-cm$^2$ plastic flasks and treated with IFN-β (300 or 3000 U ml$^{-1}$) for 24 h. Cells were harvested by vigorous pipetting and were resuspended in ice-cold buffer (PBS with 5% FBS and 0.5% sodium azide). The cells were

© Cancer Research Campaign 1998

**Figure 1** Induction of IRF-1 and IRF-2 mRNAs by IFN-β stimulation and viral infection. ONS-76 cells were treated with $10^3$ U $ml^{-1}$ IFN-β for 1 h or NDV for 1 h. Five micrograms of total RNA isolated from cells at several time intervals after each induction were subjected to Northern blot analysis. The filters were stained with methylene blue to show 28S ribosomal RNA and then probed with IRF-1 or IRF-2

incubated with 1:1000 diluted monoclonal antibody against MHC class I antigens (Bio-Rad, Richmond, CA, USA) for 30 min at 4°C and then washed three times with the buffer. The cells were then incubated in a 1:100 dilution of goat anti-mouse IgG conjugated to fluorescein isothiocyanate (Sigma, St Louis, MO, USA) in the buffer for 30 min at 4°C. Thereafter, the cells were washed three times in ice-cold PBS and analysed on an EPICS Elite flow cytometer (Coulter, Hlaleah, FL, USA).

### RESULTS

### Induction of IRF-1 and IRF-2 mRNAs in ONS-76 cells

To examine the induction of IRF-1 and IRF-2 genes in CNS-derived cells, ONS-76 cells were incubated for different time periods after IFN-β stimulation or viral infection, and levels of IRF-1 and IRF-2 mRNA were determined by Northern blot analysis. As shown in Figure 1A, after IFN-β stimulation a rapid increase for IRF-1 and IRF-2 mRNA was observed, with maximum inductions at 6 h for IRF-1 and 3 h for IRF-2. This result differed from the previous reports of fibroblasts in which IRF-1 was induced very quickly after IFN-β stimulation (Fujita et al, 1989*b*; Harada et al, 1989; Reis et al, 1992). Under NDV infection, IRF-1 and IRF-2 genes were induced more slowly than under IFN-β stimulation, and both induction levels peaked at 9 h after infection as shown in Figure 1B. This evidence was in agreement with the previous reports of fibroblasts (Fujita et al, 1989*b*; Harada et al, 1989; Reis et al, 1992). These results indicate that IRF-1 and IRF-2 mRNA were expressed by IFN-β stimulation and viral infection in CNS-derived cells as well as fibroblasts, apart from the delayed induction of IRF-1 after IFN-β stimulation.

### Generation of IRF-1 or IRF-2 overexpressing ONS-76 cells

To gain further insight to the function of IRF-1 and IRF-2 in ONS-76 cells, we generated stable transfectants of ONS-76 cells over-expressing mouse IRF-1 or IRF-2 cDNA by using recombinant retroviruses. To eliminate the effect of IRF-1 and IRF-2 caused by clonal variants, we mixed approximately $10^5$ clones and analysed the average effect of IRF-1 or IRF-2. The expression of trans-genes, mouse IRF-1 and IRF-2, was detected in R1 (mixture of IRF-1 introducing clones) and R2 (mixture of IRF-2 introducing clones) cells by Northern blot analysis (Figure 2A). We used C-76 cells (mixture of no inducing clones) as control. Then, we analysed IRF-1 or IRF-2 expression for protein level by gel-shift analysis. As shown in Figure 2B, there were two bands of IRF-1 and IRF-2, in agreement with the results in fibroblasts (Harada et al, 1989; 1990). The DNA-binding activity of IRF-1 in R1 cells was 7.3-fold and that of IRF-2 in R2 cells was 8.2-fold higher than C-76 cells when analysed using a densitometer (Fuji film, Tokyo, Japan). In this study, we detected a slower migrating band reactive to anti IRF-2 antibody that was not detected in fibroblasts (Figure 2B). There was no alternation in cell growth, morphology and cytoskeletal protein markers among parental ONS-76, C-76, R1 and R2 cells (data not shown).

### IFN-β production and expression of IFN-β gene in R1 and R2 cells

Without or with IFN-β stimulation, there were no detectable levels of IFN-β production in C-76, R1 and R2 cells using ELISA (Table 1). No detectable production of IFN-β in R1 cells after IFN treatment was in agreement with results in stable transfectants overexpressing

**Figure 2** Expression of transfected mouse IRF-1 and IRF-2 cDNAs in R1 and R2 cells. (**A**) Northern blot analysis of RNAs from R2 (lane 1), R1 (lane 2), C-76 (lane 3) and mouse fibroblast L929 (lane 4) cells. Five micrograms of total RNA was subjected to Northern blot analysis. (**B**) Autoradiogram of gel shift analysis. Cell extracts were prepared from C-76 (lanes 1–3), R1 (lanes 4–6), and R2 (lanes 7–9) cells. Twenty micrograms of protein was subjected to gel shift analysis in the presence of anti IRF-1 (lanes 2, 5 and 8) or anti IRF-2 antibodies (lanes 3, 6 and 9)

© Cancer Research Campaign 1998

**Figure 3** IFN-β expression in R1 and R2 cells. (**A**) IFN-β mRNAs in C-76, R1 and R2 cells after NDV infection. Five micrograms of total RNA isolated from the cells at several time intervals after treatments with NDV for 1 h were subjected to Northern blot analysis. The filters were stained with methylene blue to show 28S ribosomal RNA and then probed with IFN-β. (**B**) The IFN-β mRNA levels of B were quantified using densitometric analysis. - - ○ - -, C-76; -●- R1; -Δ, R2. The peak expression level of C-76 cells was assigned the value of 1.0 in each graph

IRF-1 derived from other cell types (Leblanc et al, 1990; Reis et al, 1992). Under viral induction, R1 and R2 cells produced 2.46- and 2.24-fold higher IFN-β than C-76 cells, and under poly(I): poly(C) stimulation after IFN-β priming, R1 and R2 cells produced 2.33- and 1.87-fold higher IFN-β than C-76 cells respectively (Table 1). To examine whether overexpression of IRF-1 or IRF-2 affects IFN-β production at transcriptional levels, total RNA extracted from C-76, R1 and R2 cells every 3 h after viral infection were subjected to Northern blot analysis (Figure 3A). The induction of the IFN-β gene in both R1 and R2 cells was detected 3 h earlier than that of C-76 cells, and the rate of decrease after maximal induction was slower in R1 and R2 cells than in C-76 cells using densitometric measurement (Figure 3B). However, there was no significant difference in the magnitude of maximal induction in C-76, R1 and R2 cells (Figure 3B). These results indicate that not only IRF-1 but also IRF-2 overexpression in ONS-76 cells up-regulated the expression of the IFN-β gene by viral infection or double-stranded RNA stimulation.

**Figure 4** Induction of IFN-β mRNA in C-76, R1 and R2 cells by viral infection in the presence of cycloheximide. C-76, R1 and R2 cells were treated with 100 mg ml$^{-1}$ cycloheximide, 1 h before NDV infection. Eleven hours after NDV infection, each 5 μg of total RNA was subjected to Northern blot analysis. The filters were stained with methylene blue to show 28S ribosomal RNA and then probed with IFN-β. [a]Densitometric indexes (DI) were calculated by subtracting no induction levels (*) from IFN-β mRNA levels measured using densitometry

**Figure 5** Expression of 2–5 A synthetase mRNA induced by IFN-β. (**A**) The cells were treated with $10^3$ U ml$^{-1}$ IFN-β for 1 h. Five micrograms of total RNA isolated from cells at several time intervals after IFN-β stimulation were subjected to Northern blot analysis. The filters were stained with methylene blue to show 28S ribosomal RNA and then probed with 2–5 A synthetase. (**B**) mRNA levels of 2–5 A synthetase were quantified using densitometric analysis. - - ○ - -, C-76; –●–, R1; –▲–, R2

### IFN-β expression in R1 or R2 cells in the presence of cycloheximide

To examine whether or not de novo protein synthesis is necessary for IFN-β expression after viral infection, C-76, R1 and R2 cells

*© Cancer Research Campaign 1998*

**Figure 6** Induction of cell-surface expression of MHC class I antigens by IFN-β. C-76, R1 and R2 cells were treated for 48 h without or with recombinant human IFN-β (300 or 3000 U $ml^{-1}$; Toray, Tokyo, Japan). The cells were then incubated with a mouse monoclonal antibody against MHC class I antigen. Thereafter, the cells were incubated with a goat anti-mouse antibody conjugated to FITC. Cell-surface staining was quantified using EPICS Elite Flow Cytometer. Histograms depict fluorescence profiles of uninduced (—) and IFN-induced (- - -) cells. Zero point of fluorescence intensity is normalized at that of cells treated only with a goat anti-mouse antibody conjugated to FITC

were continuously treated with 100 mg $ml^{-1}$ cycloheximide (CHX), a protein synthesis inhibitor, 1 h before viral infection. Eleven hours after viral infection, when there was no significant difference in IFN-β gene expression in C-76, R1 and R2 cells (Figure 3B), total RNAs prepared from cells were applied to Northern blot analysis. As shown in Figure 4, in the presence of CHX virally induced IFN-β expression of C-76 cells was completely suppressed. However, that of R1 or R2 cells similarly treated with CHX showed 7.5- or 2.2-fold higher IFN-β expression, respectively, than C-76 cells using densitometric measurement (Figure 4). These results indicate that pre-existing IRF-1 and IRF-2 before CHX treatment up-regulated virally induced IFN-β expression, and in particular IRF-1 was much more effective than IRF-2.

### Regulation of IFN-inducible genes in R1 and R2 cells

The possible roles of IRF-1 and IRF-2 in the transcriptional regulation of IFN-inducible genes were extensively analysed (Harada et al, 1989; Watanabe et al, 1991; Matsuyama et al, 1993). Among the IFN-inducible genes known to contain IRF-binding domains in their promoter regions are the genes for 2-5 A synthetase and MHC class I antigens. Constitutive expression of IRF-1 mRNA in the sense or antisense orientation in human fibroblasts (GM637) demonstrated enhancement or suppression of the expression of IFN-inducible genes respectively (Reis et al, 1992). In this study, by using stable transfectants overexpressing IRF-1 or IRF-2 mRNA, we found a reciprocal role of IRF-1 and IRF-2 in the regulation of the expression of these genes. The levels of 2-5 A synthetase mRNA were determined by Northern blot analysis after C-76, R1 and R2 cells were exposed for different time periods to IFN-β. As shown in Figure 5A, three bands, 3.6, 3.2 and 1.6–1.8 kb, that hybridized to the probe appeared in ONS-76 cells as well as other human cell lines (Hovanessian, 1991). Induction of 2–5 A synthetase mRNA by IFN-β was observed earlier in R1 cells than in C-76 cells, whereas it was lower in R2 cells than in C-76 cells at all time periods. The kinetics of mRNA induction in R1 cells exhibited the maximum at 6 h and then a gradual decrease; in contrast, that of C-76 and R2 cells increased more slowly (Figure 5B). On the other hand, C-76, R1 and R2 cells were treated for 24 h with 300 or 3000 U $ml^{-1}$ of IFN-β, and the cell-surface expression of MHC class I antigens by IFN-β was measured using flow cytometric analysis (Figure 6). Without IFN-β treatment, expression of MHC class I antigens was higher in R1 cells and lower in R2 cells than in C-76 cells (Table 2). With 300 U $ml^{-1}$ IFN-β treatment, expression of MHC class I antigen was elevated in C-76 or in R1 cells compared with that of the respective uninduced cells, whereas R2 cells had no significant change in the fold induction. In the presence of 3000 U $ml^{-1}$ of IFN-β, which was more markedly elevated in all the type of cells than in the case of 300 U $ml^{-1}$ IFN-β, R1 cells increased 3.73-fold more than the uninduced R1 cells. In contrast, R2 cells showed least responses to the IFN-β treatment. These results indicate that IRF-1 may function as an activator and IRF-2 as a repressor of expression of 2-5 A synthetase and MHC class I antigens in CNS-derived cells.

© Cancer Research Campaign 1998

### DISCUSSION

In the present study, CNS-derived ONS-76 cells were demonstrated to possess IFN responses as well as fibroblasts; IFN-β and IFN-inducible genes were induced by viral infection and IFN-β stimulation respectively. Furthermore, we found that the expression of IRF-1 and IRF-2 was regulated in ONS-76 cells upon viral infection and IFN-β stimulation, and that overexpression of IRF-1 and IRF-2 affected the regulation of the expression of IFN-β and IFN-inducible genes. These results suggest that both IRF-1 and IRF-2 may function as regulators of IFN responses also in the CNS. Compared with fibroblasts, the delayed induction of IRF-1 after IFN treatment (Figure 1) and the slower migrating band reactive anti-IRF-2 antibody (Figure 2B) were newly observed in our model system (Harada et al, 1990; Reis et al, 1992). Further investigation will be necessary to examine whether these phenomena are unique to CNS-derived cells. As mentioned in previous reports (Fujita et al, 1989*a*; Harada et al, 1990; Reis et al, 1992; Matsuyama et al, 1993), IRF-1 acts as a transcriptional activator of IFN-β and IFN-inducible genes, and IRF-2 acts as a repressor of IRF-mediated activation. However, in our study, overexpression of IRF-2 up-regulated IFN-β gene expression and resulted in higher production of IFN-β by viral infection (Figure 3). This evidence shows that IRF-2 as well as IRF-1 acts as a transcriptional activator of the IFN-β gene upon viral infection. That the functional change also occurred in the presence of CHX (Figure 4) implied that IRF-2 may directly convert to an activator. From our present study, it is still unclear whether the activation of the IFN-β gene by IRF-2 is only limited in CNS-derived cells. On the other hand, the human histone H4 gene FO108 has been shown to be activated by IRF-2 by using cDNA overexpression study (Vaughan et al, 1995). This evidence may suggest that there exists a possible mechanism by which IRF-2 functions as a transcriptional activator. Although the mechanism by which IRF-2 activates the IFN-β gene expression is still unclear, some possible explanations are as follows.

(1) It has been reported that IRF-2 protein is truncated during viral infection (Palombella and Maniatis, 1992), and truncation of carboxy-terminal repression domain of IRF-2 may convert the function of IRF-2 to a transcriptional activator (Yamamoto et al, 1994).
(2) It has been observed that the IFN-β gene expression is inhibited by a protein kinase inhibitor, 2-aminopurine (Zinn et al, 1988). Viral-infection may induce some modification of IRF-2 protein itself such as a phosphorylation, resulting in a functional conversion of IRF-2.
(3) A factor(s) co-operates IRF-2 and activates the IFN-β gene expression, such as IRF-2 associating molecule(s) or transcription factor(s) including IRF-1, which binds to the same *cis* elements of IFN-β gene.

In our study, gel-shift assay showed a slower migrating band that was not detected in fibroblasts (Figure 2B). The substance may be the multiple form of IRF-2, which might be involved in the converting function of IRF-2. On the other hand, virally induced IFN-β expression in C-76 cells was only detected at a very low level when de novo protein synthesis was blocked by CHX, whereas that in R1 and R2 cells significantly increased more than that in C-76 cells, particularly R1 cells that were much larger than R2 cells (Figure 4). It was previously shown that IRF-1 protein was unstable with a half-life of approximately 30 min and that IRF-2 protein was apparently stable with a half-life of more than 8 h (Watanabe et al, 1991). Assuming that IRF-1 and IRF-2 are equivalently co-operative for viral induction, different stabilities might reflect upon different amounts of virally induced IFN-β gene expression between R1 and R2 cells under CHX treatment. In either case, the function of IRF-2 as a regulator of the IFN system in CNS-derived cells is supposed to be more complex than that in fibroblasts. Further studies in the future are needed to understand the role of IRF-2 in the regulation of IFN-β gene induction.

Our overexpression assays showed that IRF-1 acted as an activator and IRF-2 acted as a repressor in the regulation of IFN-inducible gene expression (Figures 5 and 6). These results agreed with previous reports (Fujita et al, 1989*a*; Harada et al, 1990; Reis et al, 1992; Matsuyama et al, 1993). However, as shown in Figure 5A and B, 2-5 A synthetase induction by IFN stimulation in R1 cells decreased after the maximum. This suppression was a novel finding in our model system. On the other hand, embryonic fibroblasts from mice with a null mutation in the IRF-1 gene showed no significant changes in the induction of IFN-inducible genes, such as 2-5 A synthetase or double-stranded RNA-dependent protein kinase under IFN stimulation (Kimura et al, 1994). This evidence demonstrates the existence of IRF-1 independent pathway in the expression of IFN-inducible genes. In fact, IFN-inducible genes are known to be activated by a family of ISGF-3 (IFN-stimulated gene factor-3) including Stat (signal transducer and activator of transcription) proteins (Darnell et al, 1994). Many cytokines, including IFNs, exhibit a wide range of biological effects in various tissues and cells, whereas different cytokines can act on the same cells type to mediate similar effects (Weissmann and Weber, 1986; Vilcek, 1990). This functional pleiotropy and redundancy may be explained in part by the existence of diverse and shared transcription factors in cytokine signalling. Thus, we have been much concerned with the involvement of other transcriptional factors such as ISGF-3 in IFN-inducible gene expression in CNS because IFNs exhibit specific effects on neuronal cells (Calvet and Gresser, 1979; D'Arcangelo et al, 1991) and other transcriptional factors may provide a clue to a mechanism for the suppression of 2-5 A synthetase after the maximal induction (Figure 5B).

It has been identified that IRF-1 functions as a tumour suppressor (Harada et al, 1993; Tanaka et al, 1994), and that IRF-1 regulates critical target genes that regulate cell cycle and apoptosis (Tanaka et al, 1994; 1996: Tamura et al, 1995). This notion is also supported by the fact that deletion of one or both alleles of the IRF-1 gene was observed in many human leukaemia, myelodysplasia and oesophageal carcinomas (Willman et al, 1993; Ogasawara et al, 1996), and that functional loss of IRF-1 by aberrant exon skipping was observed in myelodysplasia (Harada et al, 1994). In our study, we found that IRF-1 and IRF-2 may regulate IFN responses in CNS but that overexpression of IRF-1 and IRF-2 did not effect upon cell growth and phenotypes of ONS-76 cell line (data not shown). However, it will be interesting to analyse the role of IRF-1 and IRF-2 in oncogenesis of CNS-derived cells such as medulloblastoma or glioblastoma and to investigate critical target genes of IRF-1 and IRF-2 in CNS in the near future.

### ACKNOWLEDGEMENTS

We thank Professor T Taniguchi (Department of Immunology, Tokyo University) for technical advice and critical comments. We thank Dr WJ Lee for critical reading of the manuscript. This work was supported in part by a grant-in-aid for Special Project Research, Cancer Bioscience from Japan.

© Cancer Research Campaign 1998

### REFERENCES

Calvet MC and Gresser I (1979) Interferon enhanced the excitability of cultured neurons. *Nature* **278**: 558–560

Church GM and Gilbert W (1984) Genomic sequencing. *Proc Natl Acad Sci USA* **81**: 1991–1995

D'Arcangelo G, Grassi F, Ragozzino D, Santoni A, Tancredi V and Eusebi F (1991) Interferon inhibits synaptic potentiation in rat hippocampas. *Brain Res* **564**: 245–248

Darnell JE, Kerr I and Stark G (1994) Jak-STAT pathways and transcriptional activation in response IFNs and other extracellular signaling proteins. *Science* **264**: 1415–1421

Fujita T and Kohno S (1981) Studies on interferon priming: cellular response to viral and nonviral inducers and requirement of protein synthesis. *Virology* **112**: 62–69

Fujita T, Kimura Y, Miyamoto M, Barsoumian EL and Taniguchi T (1989*a*) Induction of endogenous IFN-α and IFN-β genes by a regulatory transcription factor, IRF-1. *Nature* **337**: 270–272

Fujita T, Reis LF, Watanabe N, Kimura Y and Taniguchi T (1989*b*) Induction of the transcription factor IRF-1 and interferon-β mRNAs by cytokines and activators of second-messenger pathways. *Proc Natl Acad Sci USA* **86**: 9936–9940

Harada H, Fujita T, Miyamoto M, Kimura Y, Maruyama M, Furia A, Miyata T and Taniguchi T (1989) Structurally similar but functionally distinct factors, IRF-1 and IRF-2, bind to the same regulatory elements of IFN and IFN-inducible genes. *Cell* **58**: 729–739

Harada H, Willison K, Sakakibara J, Miyamoto M, Fujita T and Taniguchi T (1990) Absence of the type 1 IFN system in EC cells: transcriptional activator (IRF-1) and repressor (IRF-2) genes are developmentally regulated. *Cell* **63**: 303–312

Harada H, Kitagawa M, Tanaka N, Yamamoto H, Harada K, Ishihara M and Taniguchi T (1993) Anti-oncogenic and oncogenic potentials of interferon regulatory factor-1 and -2. *Science* **259**: 971–974

Harada H, Kondo T, Ogawa S, Tamura T, Kitagawa M, Tanaka N, Lamphier MS, Hirai H and Taniguchi T (1994) Accelerated exon skipping of IRF-1 mRNA in human myelodysplasia/leukemia; a possible mechanism of tumor suppressor inactivation. *Oncogene* **9**: 3313–3320

Hovanessian AG (1991) Interferon-inducible double-stranded RNA-activated enzymes: a specific protein kinase and 2′5′-oligoadenylate synthesis. *J Interferon Res* **11**: 199–205

Itoh S, Harada H, Fujita T, Mimura M and Taniguchi T (1989) Sequence of cDNA coding for human IRF-2. *Nucleic Acids Res* **17**: 8372

Kamijo R, Harada H, Matsuyama T, Bosland M, Gerecitano J, Shapiro D, Lee J, Koh SI, Kimura T, Green SJ, Mak TW, Taniguchi T and Vilcek J (1994) Requirement for transcription factor IRF-1 in NO synthetase induction in macrophages. *Science* **263**: 1612–1615

Kimura T, Nakayama K, Penninger J, Kitagawa M, Harada H, Matsuyama T, Tanaka N, Kamijo R, Vilcek J, Mak TW and Taniguchi T (1994) Involvement of the IRF-1 transcription factor in antiviral responses to interferons. *Science* **264**: 1921–1924

Leblanc JF, Cohen L, Rodrigues M and Hiscott J (1990) Synergism between distinct enhanson domains in viral induction of the human beta interferon gene. *Mol Cel Biol* **10**: 3987–3993

Maruyama M, Fujita T and Taniguchi T (1989) Sequence of a cDNA coding for human IRF-1. *Nucleic Acids Res* **17**: 3292

Matsuyama T, Kimura T, Kitagawa M, Pfeffer K, Kawakami T, Watanabe N, Kündig TM, Amakawa R, Kishihara K, Wakeham A, Potter J, Furlonger CL, Narendran A, Suzuki H, Ohashi PS, Paige CJ, Taniguchi T and Mak TW (1993) Targeted disruption of IRF-1 or IRF-2 results in abnormal type I IFN gene induction and aberrant lymphocyte development. *Cell* **75**: 83–97

Miyamoto M, Fujita T, Kimura Y, Maruyama M, Harada H, Sudo Y, Miyata T and Taniguchi T (1988) Regulated expression of a gene encoding a nuclear factor, IRF-1, that specially binds to IFN-β gene regulatory elements. *Cell* **54**: 903–913

Ogasawara S, Tamura G, Maesawa C, Suzuki Y, Ishida K, Satoh N, Uesugi N, Saito K and Satodate R (1996) Common deleted region on the long arm of chromosome 5 in esophageal carcinoma. *Gastroenterology* **110**: 52–57

Palombella VJ and Maniatis T (1992) Inducible processing of interferon regulatory factor-2. *Mol Cell Biol* **12**: 3325–3336

Pestka S, Langer AJ, Zoon K and Samuel C (1987) Interferons and their actions. *Annu Rev Biochem* **56**: 727–777

Reis LFL, Harada H, Wolchok JD, Taniguchi T and Vilcek J (1992) Critical role of a common transcription factor, IRF-1, in the regulation of IFN-β and IFN-inducible genes. *EMBO J* **11**: 185–193

Rubinstein LJ (1985) Embryonic central neuroepithelial tumours and their differentiating potential: a cytogenetic view of a complex neuro-oncological problem. *J Neurosurg* **62**: 795–805

Shiojiri S, Fukunaga R, Ichii Y and Sokawa Y (1986) Structure and expression of a cloned cDNA for human (2′-5′) oligoadenylate synthetase. *J Biochem* **99**: 1455–1464

Tamura K, Shimizu K, Yamada M, Okamoto Y, Matsui Y, Park KC, Mabuchi E, Moriuchi S and Mogami H (1989) Expression of major histocompatability complex on human medulloblastoma cells with neuronal differentiation. *Cancer Res* **49**: 5380–6384

Tamura T, Ishihara M, Lamphier MS, Tanaka N, Oishi I, Aizawa S, Matsuyama T, Mak TW, Taki S and Taniguchi T (1995) An IRF-1-dependent pathway of DNA damage-induced apoptosis in mitogen-activated T lymphocyte. *Nature* **376**: 596–599

Tanaka N, Kawakami T and Taniguchi T (1993) Recognition DNA sequence of interferon regulatory factor 1 (IRF-1) and IRF-2), regulators of cell growth and the interferon system. *Mol Cell Biol* **13**: 4531–4538

Tanaka N, Ishihara M, Kitagawa M, Harada H, Kimura T, Matsuyama T, Lamphier MS, Aizawa S, Mak TW and Taniguchi T (1994) Cellular commitment to oncogene-induced transformation or apoptosis is dependent on the transcription factor IRF-1. *Cell* **77**: 829–839

Tanaka N, Ishihara M, Lamphier MS, Nozawa H, Matsuyama T, Mak TW, Aizawa S, Tokino T, Oren M and Taniguchi T (1996) Cooperation of the tumour suppressors IRF-1 and p53 in response to DNA damage. *Nature* **382**: 816–818

Tedeschi B, Barrett JN and Keane RW (1986) Astrocytes produce interferon that enhances the expression of H-2 antigens on a subpopulation of brain cells. *J Cell Biol* **102**: 2244–2253

Valtz NL, Hayes TE, Norregaard T, Liu SM and Mckay RD (1991) An embryonic origin for medulloblastoma. *New Biol* **3**: 364–371

Vaughan PS, Aziz F, van Wijnen AJ, Wu S, Harada H, Taniguchi T, Soprano KJ, Stein JL and Stein GS (1995) Activation of a cell-cycle-regulated histone gene by the oncogenic transcription factor IRF-2. *Nature* **377**: 362–365

Vilcek J (1990) Interferons. In peptide growth factors and their receptors. In *Handbook of Experimental Pharmacology*, Sporn MA and Roberts AB (eds). Springer: New York

Watanabe N, Sakakibara J, Hovanessian AG and Taniguchi T (1991) Activation of IFN-β element by IRF-1 requires a post-transcriptional event in addition to IRF-1 synthesis. *Nucleic Acids Res* **19**: 4421–4428

Weissmann C and Weber H (1986) The interferon genes. *Prog Nucleic Acids Res Mol Biol* **33**: 251–300

Willman CL, Sever CE, Pallavicini MG, Harada H, Tanaka N, Slovak ML, Yamamoto H, Harada K, Meekwer TC, List AF and Taniguchi T (1993) Deletion of IRF-1, mapping to chromosome 5q31.1, in human leukemia and preleukemic myelodysplasia. *Science* **259**: 968–971

Wong GHW, Bartlett PF, Clark-Lewis I, Battye F and Schrader JW (1984) Inducible expression of H-2 and Ia antigens on brain cells. *Nature* **310**: 688–691

Yamada M, Shimizu K, Tamura K, Okamoto Y, Matsui Y, Moriuchi S, Park KC, Mabuchi E, Yamamoto K, Hayakawa T and Mogami H (1989) Establishment and biological characterization of human medulloblastoma cell lines. *Brain Nerve* **41**: 695–702

Yamamoto H, Lamphier MS, Fujita T, Taniguchi T and Harada H (1994) The oncogenic transcription factor IRF-2 possesses district repression and latent activation domains. *Oncogene* **9**: 1423–1428

Zinn K, Keller A, Whittemore LA and Maniatis T (1988) 2-aminopurine selectively inhibits the induction of beta-interferon, *c-fos*, and c-*myc* gene. *Science* **240**: 210–213

© Cancer Research Campaign 1998